# New paradigms for the treatment of lysosomal storage diseases: targeting the endocannabinoid system as a therapeutic strategy

**DOI:** 10.1186/s13023-021-01779-4

**Published:** 2021-03-25

**Authors:** Edward H. Schuchman, Maria D. Ledesma, Calogera M. Simonaro

**Affiliations:** 1 Department of Genetics and Genomic Sciences, Icahn School of Medicine At Mount Sinai, 1425 Madison Avenue, Room 14-20A, New York, NY 10029 USA; 2 Centro Biologia Molecular Severo Ochoa, 28049 Madrid, Spain

**Keywords:** Lysosomal storage diseases, Endocannabinoids, Treatment

## Abstract

Over the past three decades the lysosomal storage diseases have served as model for rare disease treatment development. While these efforts have led to considerable success, important challenges remain. For example, no treatments are currently approved for nearly two thirds of all lysosomal diseases, and there is limited impact of the existing drugs on the central nervous system. In addition, the costs of these therapies are extremely high, in part due to the fact that drug development has focused on a “single hit” approach – i.e., one drug for one disease. To overcome these obstacles researchers have begun to focus on defining common disease mechanisms in the lysosomal diseases, particularly in the central nervous system, with the hope of identifying drugs that might be used in several lysosomal diseases rather than an individual disease. With this concept in mind, herein we review a new potential treatment approach for the lysosomal storage diseases that focuses on modulation of the endocannabinoid system. We provide a short introduction to lysosomal storage diseases and the endocannabinoid system, followed by a brief review of data supporting this concept.

## Lysosomal storage diseases

The lysosomal storage diseases (LSDs) represent a group of over 60 inherited rare disorders, mostly due to dysfunctional lysosomal enzymes or transport proteins [1]. The result of these abnormalities is the accumulation of macromolecules, first within the lysosomes but eventually extending to other cell compartments. Over time these anomalies lead to cell dysfunction and tissue damage, including but not limited to inflammation, fibrotic changes and cell death. Although the initiating storage molecules may be different in the individual LSDs, many of the resultant cellular pathologies are similar.

The LSDs have been a model for rare disease treatment development since the late 1960s, when the pioneering work of Neufeld and colleagues first demonstrated the principle of enzyme “cross-correction” [2]. This led to a highly productive period of research where investigators defined the mechanisms by which enzymes were targeted to lysosomes, secreted, and then taken up by cells, leading to the early development of enzyme replacement therapy (ERT) for these disorders [3]. The first commercial success of ERT for Gaucher disease in the early 1990s [4], coupled with the implementation of several incentive programs by regulators to develop drugs for rare diseases, focused intense academic and pharmaceutical interest on the LSDs over the ensuing three decades, leading to many new therapies and dozens of companies engaged in drug development.

Most of these treatment efforts have focused on recovering the missing lysosomal enzyme function in patient cells, either by protein or gene replacement [5]. To date, regulatory authorities have approved fourteen ERTs (for ten individual LSDs), and several more are under development. Gene therapies have also been extensively studied in LSD animal models since the early 1990s, but only recently have advanced to clinical trials as the technologies have matured [6]. Both of these therapeutic strategies represent “single-hit” approaches, where one drug is developed for one disease. Other approved therapies include chaperone therapies directed towards recovery of misfolded lysosomal proteins [7], and substrate reduction therapy, which aims to slow the production of macromolecules using specific enzyme inhibitors [8].

Despite the success of many of these therapies, significant challenges remain. For example, although ERTs are available for several diseases, in many cases the administered proteins cannot reach important sites of pathologies effectively, including the skeletal and central nervous systems (CNS) [9]. Thus, for some diseases the ERTs are highly effective, but for others the clinical impact is limited. In fact, over half of all LSDs have an important neurologic component, and the inability of the administered enzymes to cross the blood brain barrier (BBB) is a major limitation of all ERTs. To overcome this obstacle fusion enzymes are being developed that facilitate movement of the proteins across the BBB [10], and gene therapies are being developed that directly express the enzymes in the CNS [11]. In addition, small molecules (e.g., chaperones, substrate reduction drugs) are being developed to cross the BBB [12].

Another important limitation of many of the existing or pending therapeutic approaches is their expense. For example, ERTs can cost anywhere from $250–650,000/year per patient [13], and gene therapies are being estimated to cost millions of dollars that are likely to be paid out over several years. These high costs have been justified by the research and development costs incurred by the drug companies, as well as the cost saving value of the treatments when compared to the medical costs of caring for these patients in the absence of these therapies. However, another important factor is the very small commercial market for these disorders, especially when the drugs are being developed for individual diseases.

Thus, in recent years researchers have begun to turn towards therapies that target common disease mechanisms in the LSDs, including autophagy, inflammation and others [e.g., 14–16]. Such approaches have the advantage of potentially being used in multiple diseases, thus reducing development costs and ultimately the cost to patients. They also may complement the ERT and gene therapy strategies that are being used to replace the defective proteins in individual diseases. Here we propose one such new approach that targets the endocannabinoid system (ECS), and discuss the rationale underlying this approach for the LSDs.

## The endocannabinoid system

The ECS consists of several endogenous lipid signaling molecules, the most abundant of which are anandamide (AEA) and 2-arachidonoylglycerol (2-AG), and two G-coupled protein receptors, CB1 and CB2 [17]. CB1 is the predominant ECS receptor in the CNS and plays an important role in mediating anxiety, pain and other neurologic responses [18, 19]. CB1 is also expressed at high levels on sensory nerves that innervate peripheral tissues. In contrast, CB2 is primarily expressed in non-neuronal immune cells (e.g., glial cells in the CNS; macrophages in other organs), and has been linked to the modulation of inflammation and many other cellular functions [20, 21]. It is notable that in most LSDs chronic inflammation in neural and non-neural tissues is an important component of the disease pathology.

We now know that the neural and behavioral effects of exogenously administered cannabinoids (e.g., tetrahydrocannabinol [THC], cannabidiol [CBD]) can be traced, at least in part, to the modulation of the ECS [22], although it was first described as an endogenous system controlling nervous system function. To elicit these broad effects, numerous downstream signaling events result from activation of the CB1/CB2 receptors by endogenous endocannabinoids. Based on this, manipulation of ECS signaling has been investigated for the treatment of many diseases, with the main goal being the identification of molecules that can modulate the system without the psychoactive effects attributed to THC and other exogenous molecules [e.g., 17, 23, 24]. To date, dozens of such ECS modulator molecules have been developed and numerous clinical trials have been undertaken, mostly to alleviate pain or for the treatment of anxiety disorders or other neuropsychiatric diseases.

Of direct relevance to the LSDs, endocannabinoids are known to bind and activate their receptors by lateral diffusion within the lipid bilayer, rather than through direct interactions at the cell surface [25]. Moreover, both CB1 and CB2 receptors are integral membrane proteins that function within lipid raft structures of the membrane, and changes in sphingomyelin, cholesterol and other raft lipids (e.g., ceramide and sphingosine) have important effects on their expression, distribution and function [26–28]. In many of the LSDs, abnormal accumulation of these lipids occurs, either as the primary defect or as a secondary consequence of the lysosomal dysfunction, resulting in the disruption of lipid raft signaling. Thus, it is likely that the function of CB1/CB2 receptors may be disrupted in many of the LSDs, as has been shown in a mouse model of Type C Niemann-Pick disease [29].

## The endocannabinoid system and treatment of the lysosomal storage diseases

As noted above, CB1 receptors are primarily expressed in neurons, while CB2 receptors are primarily expressed in immune cells. A large number of small molecules have been developed to modulate the function of CB1/CB2 receptors, and many of these cross the BBB. In addition, several of these molecules have been evaluated in clinical trials and safety data has been established. One of the major gaps in current treatments for the LSDs is the lack of therapies for the CNS, and therefore repurposing these ECS molecules is a logical approach. Such ECS effector molecules have been shown to reduce inflammation, slow cell death processes, improve mitochondrial, lysosomal and other cell functions, and enhance other metabolic pathways that are commonly defective in the LSDs (Table 1).

**Table 1:** Common cell processes defective in LSDs that may be impacted by CB1/CB2 modulator drugs

Cell function	Reference example
Autophagy	[39]
Calcium homeostasis	[40]
Cell survival/death	[41]
Endo/exocytosis (vesicular transport)	[42]
Endothelial cell function (BBB integrity)	[43]
Extracellular matrix production/fibrosis	[44]
Inflammation	[21]
Mitochondrial function/energy balance	[45]
Myelination	[46]
Synaptic function	[47]

As an example, an early paper showed that activation of CB1 receptors with the endocannabinoid AEA or with THC, the main psychoactive compound in cannabis, led to sphingomyelin degradation in cultured astrocytes through activation of a neutral sphingomyelinase [30]. In the LSD acid sphingomyelinase deficient Niemann-Pick disease (i.e., types A and B Niemann-Pick disease, ASMD), sphingomyelin storage is the primary metabolic abnormality [31], suggesting that CB1 activation may be a reasonable approach for the treatment of this disorder. Importantly, although the sphingomyelin storage in ASMD begins in lysosomes, it rapidly extends to the plasma membrane, mitochondria and other cell compartments, making it potentially accessible to the neutral sphingomyelinase activity [32]. Although an ERT is in the final stages of clinical development for this disorder (Olipudase alfa®), it does not impact the CNS disease that occurs in about half of all ASMD patients [33].

We therefore decided to evaluate this approach in a mouse model of type A and B NPD (acid sphingomyelinase deficient mice, ASMKO) [34], and found the unexpected downregulation of CB1 on the surface of neurons from these mice [35]. This was due to entrapment of the receptor within lysosomes, indicating that CB1 signaling was likely abnormal in this disorder and further supporting the activation of CB1 as a therapeutic strategy. Since direct activation of CB1 with synthetic or natural agonists leads to psychotropic effects, we chose an indirect strategy using small molecules that inhibit the enzyme fatty acid amide hydrolase (FAAH), leading to an elevation of endogenous endocannabinoids, including AEA, and several other bioactive lipids (e.g., palmitoylethanolamide [PEA] and oleoylthanolamide [OEA]). AEA primarily acts on CB1 receptors and to a lesser extent CB2, while PEA and OEA act on the PPAR-α receptor. Many FAAH inhibitors have been developed that cross the BBB, and several have entered clinical trials [23].

**Fig. 1:**
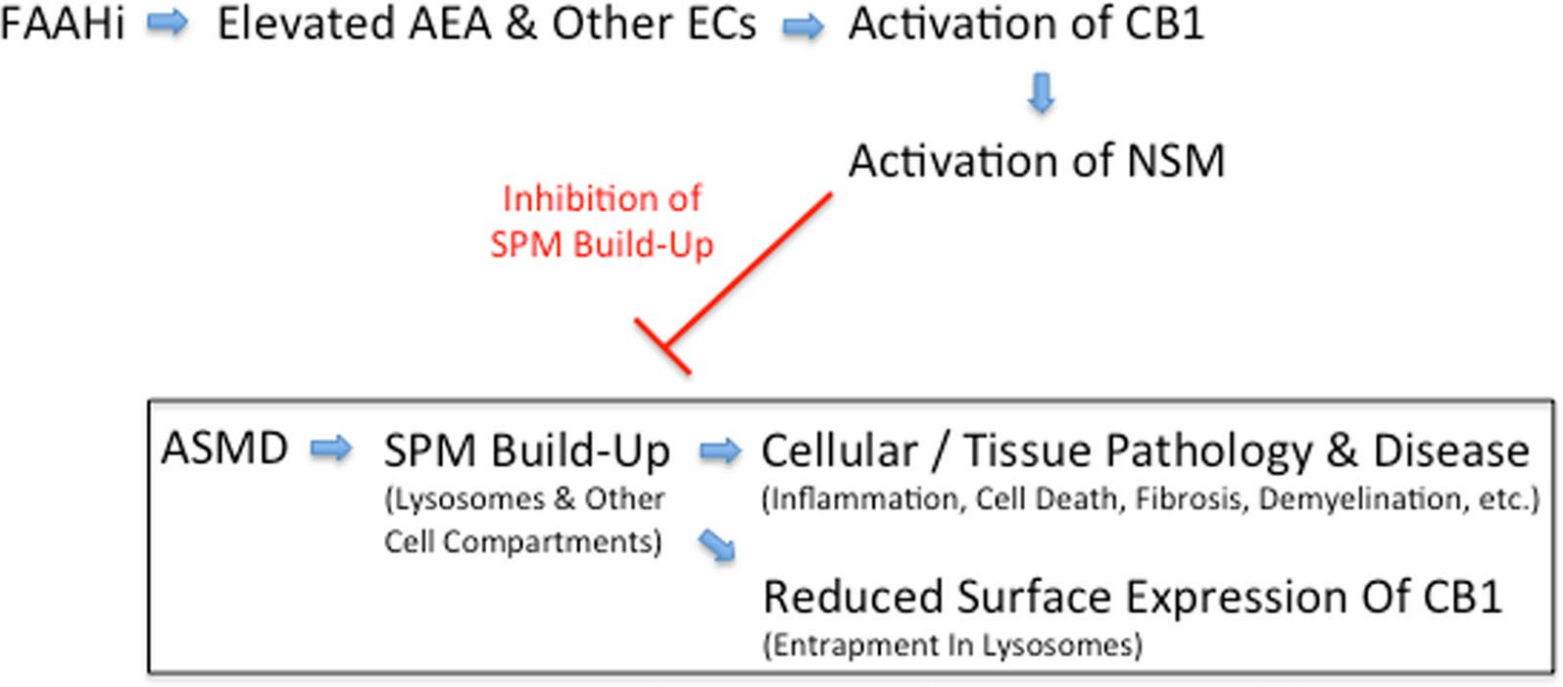
Effect of FAAH inhibition in ASMD. All pathology in ASMD is initiated by sphingomyelin (SPM) build-up. FAAH inhibition leads to the elevation of AEA and other endocannabinoids (ECs), resulting in the activation of CB1. This, in turn, activates neutral sphingomyelinase (NSM), which slows or prevents SPM buildup and the resulting downstream pathology and disease

We first evaluated the ability of three such FAAH inhibitors to reduce sphingomyelin levels in neurons from ASMKO mice. Significant sphingomyelin reduction was observed, which could be prevented using an inhibitor of neutral sphingomyelinase [35]. We then treated ASMKO mice with one FAAH inhibitor, and found improvements in many pathologic markers, including reduction of sphingomyelin in the brain and other tissues. Importantly, several neurological clinical endpoints also were improved, and lifespan was significantly extended as well. A schematic depiction illustrating the putative mechanism of action of FAAH inhibition in ASMD is shown in Fig. 1. We also found that treatment of cells from patients with type C Niemann-Pick disease, due to mutations in the *NPC1* gene, led to reduction of sphingomyelin and, importantly, reduction in cholesterol storage. This is consistent with previous work showing that reduction of sphingomyelin in type C Niemann-Pick disease cells using recombinant acid sphingomyelinase led to correction of cholesterol trafficking [36]. While these examples may be specific to diseases with significant sphingomyelin storage, activation of CB1 receptors has many other beneficial effects that could impact all LSDs, including prevention of neurodegeneration, inhibition of pain, remyelination and others.

In contrast to CB1, CB2 receptors are primarily expressed on immune cells both in the periphery and CNS. Therefore, unlike CB1, direct agonists of CB2 receptors do not have major psychotropic effects, and have been safely used in animal models of various diseases. The main effect of these direct CB2 agonists is to reduce inflammatory responses in autoimmune and other inflammatory diseases, including neuroinflammatory diseases, although additional positive effects on prevention of cell death, repair of the BBB, and other cell abnormalities have been reported [20, 21].

**Fig. 2:**
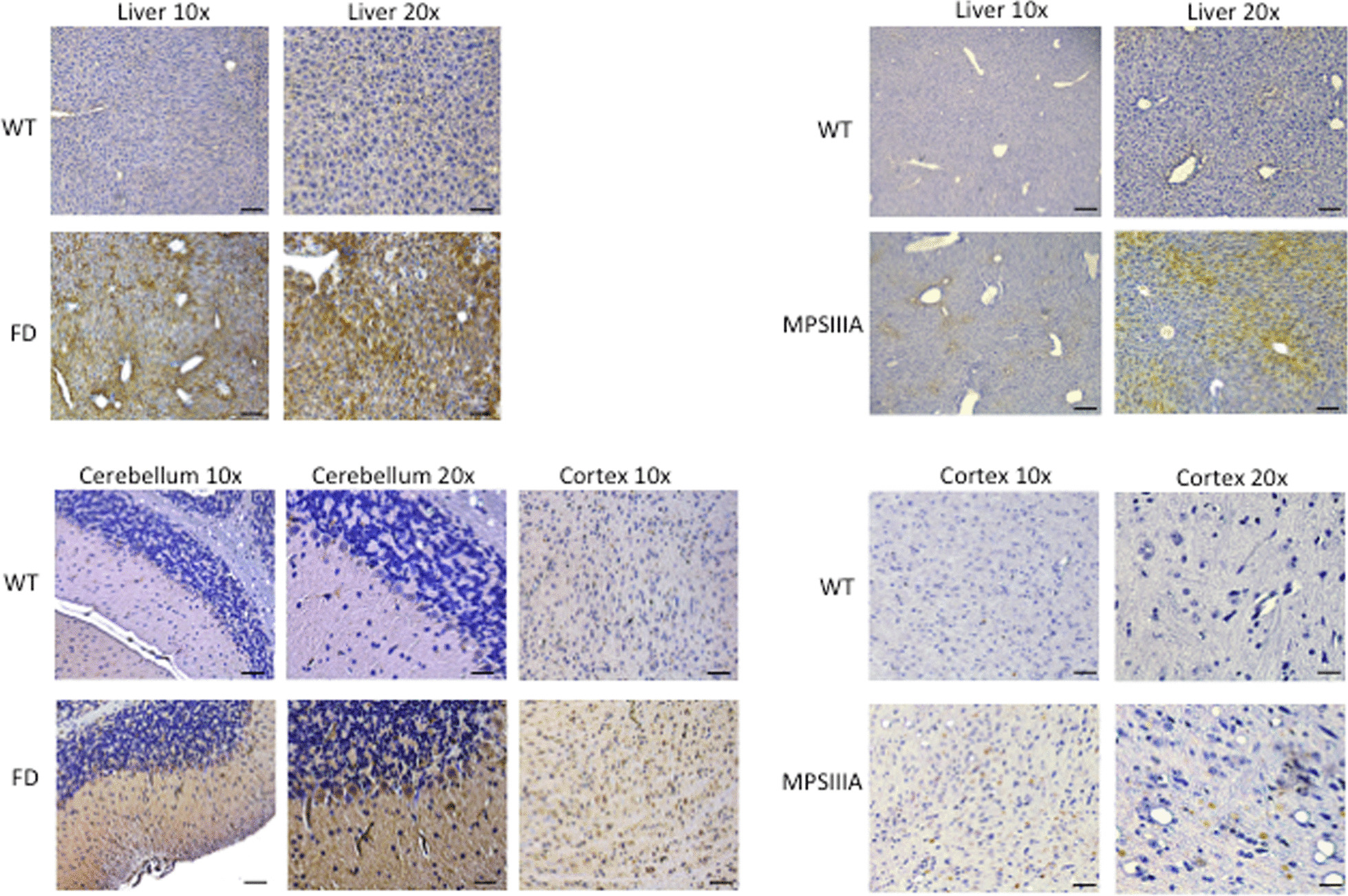
CB2 expression in tissues from Farber and MPS IIIA mice. CB2 is markedly overexpressed in the liver and CNS of mice with Farber disease and MPS IIIA. WT, wildtype; FD, Farber. Brown color indicates staining with CB2 antibodies. Blue color indicates cell nuclei. Bars = 50 µm

To initially evaluate the role of CB2 in the LSDs, we chose mouse models for two diseases: acid ceramidase deficiency (Farber disease) and mucopolysaccharidosis type IIIA (Sanfilippo A; MPS IIIA) [37, 38]. Although the initiating storage material in these two diseases is distinct (the lipid ceramide and the glycosaminoglycan heparan sulfate, respectively), both of these disorders are characterized by early activation of inflammatory pathways in the periphery and CNS. Consistent with this inflammatory disease, CB2 expression was found to be markedly elevated in several tissues from these animals (Fig. 2). Treatment studies using a CB2 agonist are underway, but preliminary findings have already revealed a slowing of disease progression and extension of lifespan in the Farber disease animals, consistent with the reduction of inflammation and other positive effects attributed to these molecules. Thus, CB2 receptors are not only a potential new target for treatment, but also may serve as a biomarker to indicate macrophage infiltration and inflammation in different LSDs.

## Concluding thoughts

We propose that the ECS represents a new and potentially important target for the treatment of some LSDs. The fact that many molecules that modulate this system have already been developed, some of which cross the BBB and are active in the CNS, should facilitate these repurposing efforts. Preliminary evidence in several LSD animal models indicates the potential of this approach, but significant questions still remain. Among them is the fact that most studies to date using CB1/CB2 modulator drugs in animal models or clinical trials have evaluated them for relatively short periods of time (days or weeks). In the case of chronic diseases such as the LSDs, long-term treatment will be required, and the safety of these molecules must be established in this context, along with the proper dosing. The psychotropic effects and potential for dependency attributed to some of these molecules also indicates the need for more evaluation in the LSD models, and could especially limit their use in young children. This is a specific concern for CB1 activation, although FAAH inhibition may overcome this obstacle.

As the LSD field moves forward into the next decade, researchers and clinicians must develop a new paradigm for LSD drug development that builds upon the substantial progress that has already been made. Such drug development must address the missing needs of the current therapies, such as targeting difficult to reach pathologic organs including the CNS and skeletal system, and also must move away from the “single-hit” approach to drug development in an effort to make the process more efficient and ultimately the costs of these drugs less prohibitive. We are currently witnessing the early investigation of these broader approaches, and expect that further studies of this nature, including investigation of the ECS system, will continue to offer new hope to LSD patients.

## Data Availability

All data are included in this published article and reference [35].

## Abbreviations

AEA: Anandamide
2-AG: 2-Arachidonoylglycerol
ASMD: Acid sphingomyelinase deficient Niemann-Pick disease
ASMKO: Acid sphingomyelinase knock-out
BBB: Blood brain barrier
CBD: Cannabidiol
CNS: Central nervous system
ECS: Endocannabinoid system
ERT: Enzyme replacement therapy
FAAH: Fatty acid amide hydrolase
LSDs: Lysosomal storage diseases
MPS: Mucopolysaccharidosis
THC: Tetrahydrocannabinol
